# The effect of coenzyme Q10 pretreatment on ovarian reserve in women undergoing hysterectomy with bilateral salpingectomy: a randomised, double-blind, placebo-controlled trial

**DOI:** 10.1017/S0007114524003131

**Published:** 2025-01-28

**Authors:** Kanockpetch Micaraseth, Siriluk Tantanavipas, Woradej Hongsakorn, Artitaya Singwongsa

**Affiliations:** 1 Department of Obstetrics and Gynaecology, Faculty of Medicine, Ramathibodi Hospital, Mahidol University, Bangkok 10400, Thailand; 2 Reproductive Endocrinology and Infertility Unit, Department of Obstetrics and Gynaecology, Faculty of Medicine, Ramathibodi Hospital, Mahidol University, Bangkok, Thailand

**Keywords:** Ovarian reserve, Hysterectomy, Coenzyme Q10, Ubiquinol

## Abstract

The effect of diminished ovarian reserves after undergoing hysterectomies with bilateral salpingectomies is one of the health concerns among reproductive-age women with benign gynecological diseases. Coenzyme Q10 (CoQ10), an antioxidant, is crucial in mitochondrial energy production, improving oocyte quality and quantity. This study compares the benefit of a 14-d preoperative (CoQ10) *v*. placebo on ovarian reserve by measuring anti-Müllerian hormone (AMH) in women undergoing hysterectomy with bilateral salpingectomy. A double-blinded, randomised, placebo-controlled trial was conducted. Forty-four women with benign gynecological diseases were randomised to receive either oral CoQ10 300 mg per d or placebo for 14 d before undergoing hysterectomy with bilateral salpingectomy. Serum AMH levels were collected for analysis before taking CoQ10 and 6 weeks postoperatively in each group. The baseline demographic, clinical characteristics and baseline AMH levels were comparable between the groups (1·47 (0·45, 2·49) *v*. 1·29 (0·47, 2·11), *P* = 0·763). The serum AMH levels after the surgery were significantly decreased from preoperative levels (median 0·99 (0·37, 1·63) *v*. 1·34 (0·57, 2·30)), *P* = 0·001. However, there was no significant difference in the AMH change between the CoQ10 group and the placebo group (AMH per cent change −28·2 % (64·09, −4·81) *v*. −20·07 % (–61·51, −2·92)), *P* = 0·99, respectively. Age, gynecological disease, operative time and blood loss were not significantly associated with the AMH change. There were no significant side effects or adverse operative outcomes among CoQ10 users. In conclusion, hysterectomy with bilateral salpingectomy led to a significantly decreased AMH level. However, pretreatment with CoQ10 for 2 weeks was ineffective in protecting an ovarian reserve.

Hysterectomy with bilateral salpingectomy is the most common gynecological surgical procedure performed for several benign gynecological diseases^(1,2)^. Owing to the recognition that the fallopian tubes are the site of origin for high-grade serous ovarian cancers, opportunistic bilateral salpingectomy has increased significantly since 2010 to lower the risk of ovarian cancer^(3,4)^. Nevertheless, there is a concern regarding the possible risk of diminished ovarian reserves from decreased blood supply^(5–8)^. Overall damage could result at an earlier age of menopause^(6,9)^, often associated with unpleasant symptoms, including vasomotor symptoms, genitourinary symptoms and increased risk of osteoporosis and CVD^(9,10)^. Some studies demonstrated that ovarian-sparing hysterectomy showed a significant decline in anti-Müllerian hormone (AMH) levels and a higher proportion with undetectable levels^(7,8,11)^. Moreover, the meta-analysis demonstrated that postoperative AMH in women undergoing hysterectomy with bilateral salpingectomy was 0·94 ng/ml (95 % CI –1·89, 0·01) lower than hysterectomy alone^(12)^.

Coenzyme Q10 (CoQ10), or Ubiquinone, is a lipid-soluble endogenous antioxidant. It is well known for its role in the electron transport chain in mitochondrial membranes during aerobic cellular respiration^(13–15)^. Besides antioxidative functions, CoQ10 can also decelerate cell degeneration^(16)^. Numerous disease processes associated with CoQ10 deficiency can benefit from CoQ10 supplementation, including primary and secondary CoQ10 deficiencies, mitochondrial diseases, fibromyalgia, CVD, neurodegenerative diseases, cancer and infertility^(16,17)^.

In animal studies, CoQ10 supplementation protected ovarian tissue from chemotherapy-induced oxidative stress^(18,19)^. Furthermore, human studies showed that CoQ10 pretreatment in women undergoing assisted reproductive technology can improve ovarian response^(20–22)^. They found that the intervention group has improved ovarian response to stimulation, increased clinical pregnancy and live birth rates per embryo transfer^(20,22)^. On the other side, CoQ10 therapy before cardiac surgery increased myocardial tolerance to hypoxic stress, reduced inotropic drug requirements after surgery and reduced the incidence of ventricular arrhythmia^(23,24)^. During a hysterectomy, oxidative free radicals are produced during the decreased ovarian blood supply^(25,26)^. Consequently, CoQ10 pretreatment may preserve ovarian function during the procedure.

However, there is limited data on the protection of the ovarian reserve after hysterectomy with bilateral salpingectomy. This study assessed the benefit of CoQ10 supplementation before hysterectomy with bilateral salpingectomy in the ovarian reserve by measuring AMH.

## Objectives

The primary objective was to compare the benefit of 14-d preoperative CoQ10 on ovarian reserve in women undergoing hysterectomy with bilateral salpingectomy.

The secondary objectives were to determine operative factors associated with a decreased ovarian reserve and side effects of 14 d of the CoQ10 supplement.

## Material and methods

### Study design and randomisation

This was a double-blind, randomised, placebo-controlled trial study conducted between September 2022 and July 2023 in Ramathibodi Hospital, Mahidol University, Thailand. Informed consent was obtained from all participants before entering this study. The study was ethically approved by the Human Research Ethics Committee, Faculty of Medicine Ramathibodi Hospital, Mahidol University (MURA2022/185), and registered (Thai clinical trial registry; TCTR20230926003).

The women with benign gynecological conditions were recruited. The inclusion criteria were women with leiomyoma or adenomyosis scheduled for hysterectomy with bilateral salpingectomy who provided signed informed consent. The participants with a history of ovarian surgery, anticipated ovarian cystectomy, hormonal use within 3 months before surgery,

Known allergies to CoQ10 or starch suspected malignancy or use of drugs that interact with CoQ10 (i.e. warfarin and statin) were excluded from the study.

**Figure 1.**
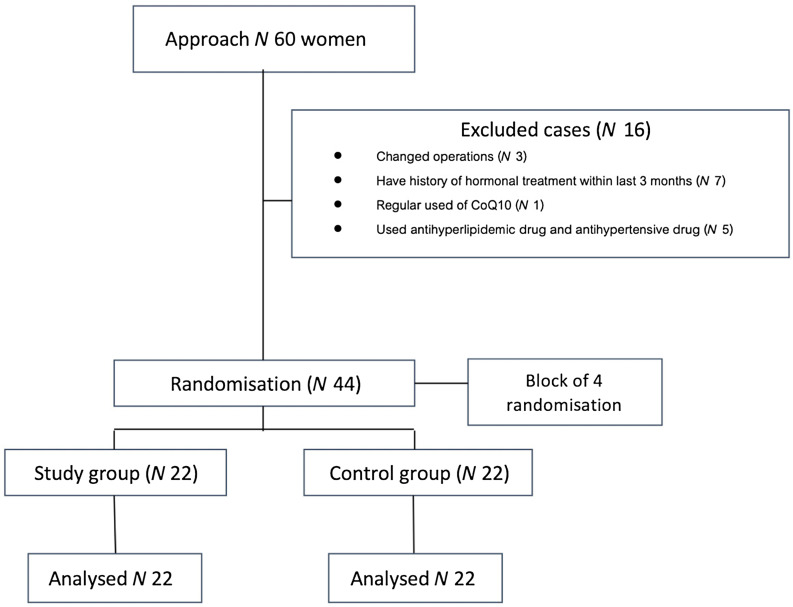
The flow of the participants through the trial. CoQ10, coenzyme Q10.

The study reported following CONSORT guidelines (Fig. 1). All participants who provided informed consent were computerised-randomised (blocks of 4) to receive either oral CoQ10 or placebo. All participants were prohibited from using other supplements and vitamins during the study period. The study participants and investigators were blinded to the patient grouping. The questionnaires were complete, including age, demographic information, BMI, parity, underlying disease, clinical indication for hysterectomy and previous abdominal surgery.

Blood samples were collected for analysis in two periods: preoperative (baseline) before taking Coq10 (or placebo) and postoperatively at 6 weeks. Serum AMH levels were measured using an automatic chemiluminescence immunoassay on Cobas e801 module (Roche Diagnostics). The limit of detection was reported as < 0·01 ng/dl. Intra-assay and inter-assay coefficients of variation were 8 % and 12 %, respectively.

### Interventions

The intervention group received oral CoQ10 300 mg oral administration daily for 14 d before surgery. The control group received identical soft capsules. All supplement packages were similar, with labels ‘A’ and ‘B’ on the bottles. Compliance with treatment was evaluated for each participant by telephone 1 week later and pill count at the end of the 2-week trial.

### Outcome measures

The primary outcome measure was a serum AMH level 6 weeks after hysterectomy with bilateral salpingectomy between the two groups.

Secondary outcomes included clinical side effects of CoQ10 (nausea, vomiting, abdominal discomfort, headache or skin rash) together with serum creatinine and liver function test, operative factors that may be associated with decreased ovarian reserve such as operative time, blood loss, adhesion and other abnormal findings were recorded.

### Sample size

The sample size calculation for this study was based on a decrease in mean serum AMH level after hysterectomy. According to the study in 2012, premenopausal hysterectomised women had a decline of mean serum AMH levels of about 0·84 ng/ml, with a sd of 0·9^(27)^. We assumed that pretreatment CoQ10 would stabilise the AMH level before surgery with α 0·05 and power 80 %. When accounting for a dropout rate of 20 %, each group required twenty-two participants.

### Statistical analysis

Student’s *t* test or Mann–Whitney *U* test was used to compare continuous variables. The normality test was done using the Shapiro–Wilk test. A non-parametric test was used for the non-normally distributed data. The *χ* ^2^ or Fisher’s exact test was used for categorical comparative variables. Descriptive results were presented as mean (sd), median and interquartile range (IQR) or as a percentage. A median regression analysis was performed to determine the possible factors associated with decreased ovarian reserve. Statistical analysis was performed using Stata Statistical Software, version 17.0 (StataCorp LLC). The statistical significance was set at a *P* value < 0·05 with a 95 % CI.

## Result

A total of sixty women met the inclusion criteria. Sixteen participants were excluded, and three undergoing changed operations, including salpingo-oophorectomy and myomectomy. These resulted in forty-four participants being enrolled in the study and randomised to twenty-two participants in each group. Both groups’ baseline characteristics and preoperative AMH values were comparable (Table 1) – the means BMI of the women in both groups tended to be overweight. All participants demonstrated complete adherence to the prescribed supplements under investigation. None of the participants who had been diagnosed with dyslipidemia were taking any lipid-lowering drugs. The most common indication for surgery was myoma uteri (*n* 32, 72·73 %), as defined by the final pathological diagnosis. The main operative procedures were similar between the two groups, with laparoscopic total hysterectomy accounting for 13·64 % (*n* 6). Concomitant surgeries were comparable. Preoperative serum AMH levels and antral follicle count were not significantly different between the two groups. The baseline serum AMH levels had a positive correlation with antral follicle count according to the Pearson correlation formula (R^2^ = 0·49)

**Table 1.** Baseline characteristics of the study population (Numbers and percentages; median values and interquartile ranges)

	Study group (*n* 22)		Control group (*n* 22)		
Characteristics	*n*	%	*n*	%	*P*
Age (years)					
Median	41·50		42		1·00
IQR	39·5, 43·5		39, 45		
BMI (kg/m^2^)					
Median	23·25		24·73		1·32
IQR	18·80, 27·69		21·91, 27·55		
Parity, *n* (%)					
0	15	68·18 %	9	40·91 %	0·48
1	1	4·55 %	5	22·73 %	
≥ 2	6	27·27 %	8	36·36 %	
History of tubal ligation, *n* (%)	5	22·73 %	7	31·82 %	0·50
Medical co-morbidities, *n* (%)					
Dyslipidemia	2	9·09 %	1	4·55 %	0·46
Heart disease	1	4·55 %	0		
Allergic rhinitis	1	4·55 %	3	13·67 %	
Migraine	0		1	4·55 %	
HIV	1	4·55 %	0		
Others	1	4·55 %	2	9·09 %	
Menarche (year)					
Median	13		13		0·754
IQR	11·5, 14·5		12, 14		
Gynecological disease, *n* (%)					
Myoma uteri	16	72·73 %	16	72·73 %	0·51
Adenomyosis	2	9·09 %	4	18·18 %	
Myoma with adenomyosis	4	18·18 %	2	9·09 %	
Surgical history, *n* (%)					
Cesarean section	6	27·27 %	6	27·27 %	1·0
Myomectomy	5	22·73 %	0		0·02
Appendectomy	2	9·09 %	3	13·64 %	0·64
Others	1	4·55 %	1	4·55 %	1·0
Smoking, *n* (%)	1	4·55 %	0		0·31
Preoperative AMH levels (ng/dl)					
Median	1·47		1·29		0·763
IQR	0·45, 2·49		0·47, 2·11		
Preoperative AFC (follicle)					
Median	7		8		0·85
IQR	4, 10		4·5, 11·5		
Major procedures, *n* (%)					
TAH with BS	20	90·9 %	18	81·82 %	0·39
TLH with BS	2	9·09 %	4	18·18 %	
Concomitant surgery, *n* (%)					
US ligament fixation	1	0·54 %	0		0·39
Endometriosis excision	0		1	0·54 %	
Lysis adhesion	1	0·54 %	1	0·54 %	
Repaired bowel serosa	1	0·54 %	0		

AMH, anti-Müllerian hormone; AFC, antral follicle count; BS, bilateral salpingectomy; IQR, interquartile range; TAH, total transabdominal hysterectomy; TLH, total laparoscopic hysterectomy.

Statistical analysis: Student’s *t* test or Mann–Whitney *U* test (for the non-normally distributed data) was used to compare continuous variables. The *χ* ^2^ or Fisher’s exact test was used for categorical comparative variables.

**Figure 2.**
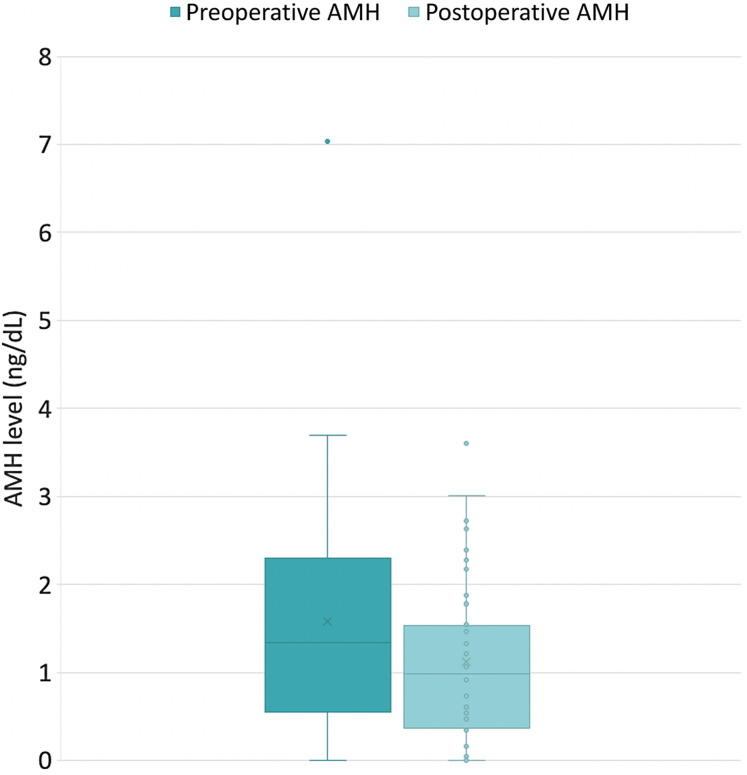
Change in serum levels of AMH after surgery 6 weeks regardless of the intervention. AHM, anti-Müllerian hormone.

According to the change in AMH at 6 weeks after the operation (Table 2), the AMH levels were decreased significantly in all participants (1·34 (0·57, 2·30) *v*. 0·99 (0·37, 1·63); *P* < 0·001) (Fig. 2). Nevertheless, there was no significant difference between the study group and the control group −0·26 ng/dl (–1·0, −0·06) and −0·13 ng/dl (–0·71, 0·02), respectively (*P* = 0·76). The percent changes were also not significantly different (*P* = 0·99). This result is the same in subgroup analysis of participants with preoperative AMH levels more than or equal to 0·05 ng/dl (*n* 17 per group).

**Table 2.** Longitudinal change in serum AMH levels (Median values and interquartile ranges)

	Study group (*n* 22)		Control group (*n* 22)		
Characteristics	Median	IQR	Median	IQR	*P*
Preoperative AMH levels (ng/dl), median (IQR)	1·47	0·54, 2·40	1·29	0·69, 2·19	0·77
Postoperative AMH levels (ng/dl), median (IQR)	0·99	0·38, 1·48	0·99	0·36, 1·88	0·71
Change in AMH change (%), median (IQR)	–28·17	–64·09, −4·81	–20·07	–61·51, −2·92	0·99
Absolute AMH change (ng/dl), median (IQR)	–0·26	–1·0, −0·06	–0·13	–0·71, 0·02	0·76

AMH, anti-Müllerian hormone; IQR, interquartile range.

Statistical analysis: the Mann–Whitney *U* test was used.

Regarding the operative factors (Table 3), both groups had comparable uterine weight, uterine volume, operative time and estimated blood loss, indicating equal levels of surgical difficulty. The uterine volume was calculated using the ellipsoid volume formula (longitudinal diameter × AP diameter × transverse diameter × 0·52). Median regression analysis was performed to determine the association of operative factors with the decrease in ovarian reserve. The data in Table 4 show that all operative variables were not significantly associated with decreasing AMH levels.

**Table 3.** Operative variables and adverse events between the two study groups (Numbers and percentages; median values and interquartile ranges; mean values and standard deviations)

	Study group (*n* 22)		Control group ( *n* 22)		
Characteristics	Median	IQR	Median	IQR	*P*
Uterine volume (cm^3^), median (IQR)	313·65	114·25, 513·06	422·29	39·78, 422·29	0·37
Uterine weight (gram), median (IQR)	292·75	106·05, 479·75	384·25	83·21, 685·29	0·37
Operative time (h), median (IQR)	2·27	1·61, 2·93	2·31	2·01, 2·61	0·763
Estimate blood loss (ml), median (IQR)	100	–25, 225	300	187·5, 412·5	0·068
Length of hospital stay (days), median (IQR)	5·00	0	5·00	4·5, 5·5	1·0
	*n*	%	*n*	%	
Adverse events, *n* (%)					
Headache	1	4·5 %	0		0·31
Nausea	1	4·5 %	0		
Diarrhoea	1	4·5 %	0		
Postoperative laboratory					
Creatinine (mg/dl)					
Mean	0·65		0·64		0·102
sd	0·11		0·19		
	Median	IQR	Median	IQR	
BUN (mg/dl), median (IQR)	9	7·5, 10·5	10	7·91, 12·09	0·794
AST (U/L), median (IQR)	22	18·25, 25·75	24	17·5, 30·5	0·354
ALT (U/L), median (IQR)	20·5	12·88, 28·13	24·5	14·25, 34·75	0·757

ALT, alanine aminotransferase; AST, aspartate aminotransferase; BUN, blood urea nitrogen; IQR, interquartile range.

Statistical analysis: the Mann–Whitney *U* test was used to compare continuous variables. Fisher’s exact test was used for categorical comparative variables.

**Table 4.** Median regression analysis of AMH change and associated factors (Coefficients and 95 % confidence intervals

Factors	Coefficient	95 % CI	*P*
Age	–0·01	–0·06, 0·04	0·665
BMI	0·00002	–0·04, 0·04	0·999
Type of operation	0·168	–0·335, 0·671	0·504
Blood loss	0·0001	–0·0004, 0·0006	0·640
Operative time	–0·19	–0·41, 0·03	0·093
Duration of hospital stay	–0·08	–0·36, 0·19	0·538
Uterine weight	–0·0002	–0·0003, 0·0007	0·462

AMH, anti-Müllerian hormone.

Statistical analysis: median regression analysis.

In the CoQ10 group, the adverse events occurred in two patients: one patient had a headache with nausea and another had diarrhoea, but the symptoms were mild. There were no serious events leading to drug cessation or hospital admission. At a 6-week follow-up, participants’ complaints were non-specific to menopausal symptoms such as myalgia, dry skin and palpitation. However, all postoperative renal and hepatic enzymes were normal.

## Discussion

This study confirmed that women undergoing hysterectomy with bilateral salpingectomy had a significant decline of ovarian reserve, as determined by serum AMH level 6 weeks after surgery (mean change 0·35 ng/ml or 26·12 %, *P* < 0·01). However, pretreatment with CoQ10 for 2 weeks was ineffective in protecting an ovarian reserve. To our knowledge, this is the first study that evaluated the impact of an antioxidant supplement to protect ovarian reserve in hysterectomised women. Previous studies that used AMH levels studied ovarian reserve loss after hysterectomy with or without bilateral salpingectomy^(6,28,29)^. Diversity in the timing of follow-up, the results were varied.

Nevertheless, our study had a follow-up time of 6 weeks, according to Yuan *et al.* ^(6)^. They reported the earliest follow-up time at 6 weeks after the hysterectomy with a significant decrease in serum AMH and an increase in serum follicle-stimulating hormones (FSH).

For 6 weeks after surgery, although we found a significant decrease in AMH level, participants did not have any specific menopausal symptoms such as hot flush, vaginal dryness, insomnia or joint pain. The symptoms may appear with a longer follow-up time because women with low AMH levels have an increased risk of early menopause^(30,31)^. Another study reported vasomotor symptoms after 12 weeks of hysterectomy (18·2 %) but not correlated with significantly diminished AMH levels^(28)^.

CoQ10 is a lipid-soluble antioxidant located in inner mitochondria^(13,14)^. The possible effects of CoQ10 on ovaries are to rescue follicles from apoptosis or enhance primordial follicle activation^(13,32)^. CoQ10 has been associated with an improvement in ovarian reserve. In the rodent study, CoQ10 was found to reverse oxidative stress on ovarian tissue by increasing the number of developing follicles and stabilising the follicular structure after exposure to the chemotherapy agent^(18,19)^. CoQ10 has been used in human reproduction to improve ovarian response in a controlled ovarian stimulation cycle^(20–22,33)^. Xu Y *et al.* evaluated the benefit of CoQ10 in the poor ovarian response group. These authors reported that CoQ10 pretreatment increases the ovarian response to stimulation and improves the quality of oocytes and embryos^(20,22)^.

On the contrary, our result cannot find an improvement in ovarian reserve with 2 weeks of CoQ10 pretreatment in women undergoing hysterectomy with bilateral salpingectomy. There are a few possible explanations for our result. First, the dose and duration of CoQ10 to protect ovarian reserve are unknown. Taking CoQ10 for a sufficient period is essential to see a sustained change. Continuation of the supplement during the postoperative period may emphasise the protective effect on ovarian function. However, the participants were presented with gynecological symptoms that needed to be treated, such as heavy menstrual bleeding and pelvic pressure. The schedules for hysterectomy were made after the initial visit within 2–4 weeks. The presurgical treatment period should not be prolonged, considering the patients’ benefit. Our study used the same dose and duration of CoQ10 as Rosenfeldt *et al.* ^(23)^. They demonstrated that CoQ10 levels after 2 weeks of therapy increased sufficiently in serum and mitochondria, approximately 4 and 2·4 times greater than placebo. In addition, there was a randomised controlled trial studying the effect of sublingual CoQ10 400 mg for 7 d in traumatic mechanical ventilated patients admitted to the intensive care unit. Blood malondialdehyde and IL-6 concentrations were significantly reduced in the CoQ10 group (*P* < 0·001)^(34)^. Another randomised controlled trial in 2021 on participants administering Coq10 200 mg or placebo for 2 weeks before undergoing strenuous exercise showed that the Coq10 group had significantly improved biomarkers of bone formation^(35)^.

In contrast, a randomised controlled trial of CoQ10 pretreatment on women with poor ovarian reserve undergoing controlled ovarian stimulation showed that 600 mg of CoQ10 for 2 months was significantly beneficial in an increased number of retrieved oocytes, higher fertilisation rate and more high-quality embryos^(20)^. However, the optimal dosage and duration of CoQ10 to protect the ovarian reserve from operative damage remains unknown. The duration of CoQ10 treatment may have needed to be longer for ovarian protection.

Second, CoQ10 may not effectively protect AMH levels in all women. Owing to the CoQ10 status variation in the general population, the treatment effect may be prominent in CoQ10-deficit women^(36)^. Finally, mechanisms of decreased ovarian reserve are ovarian blood supply disruption and increased oxidative stress, but there may be other causes that antioxidants cannot protect.

Regarding the safety profile in our study, mild adverse events were similar to the available data^(13,15)^. In general, adverse effects of CoQ10 were reported < 1 %, including gastrointestinal effects, headache and allergic skin^(15)^. Our study also evaluated liver enzyme and kidney function in the postoperative period because of the CoQ10 elimination pathway through bile, urine and faeces^(15)^. The laboratory results confirmed that there was no hepatotoxicity or renal toxicity.

Despite the lack of a significant effect on AMH levels in our study, it is essential to note that CoQ10 is a generally safe and well-tolerated supplement^(13,15)^. It is also important to note that CoQ10 has other health benefits, such as improving cardiovascular health and reducing the risk of certain chronic diseases^(15–17)^. Therefore, there may be other reasons why patients who are undergoing hysterectomy with bilateral salpingectomy may want to consider taking CoQ10.

The main strength of this study is its randomised, double-blind, placebo-controlled trial design. Moreover, we observed all adverse effects, including monitoring postoperative laboratories that might be affected by the intervention. The present study’s limitations included the unknown optimal CoQ10 dose and duration. The short-term postoperative follow-up in which menopausal symptoms may not typically present. Moreover, this study did not control lifestyle factors, such as dietary and physical activity, that could affect the outcome. Further research is needed to determine the optimal dose and duration of CoQ10 treatment for protecting ovarian reserve in women undergoing hysterectomy with bilateral salpingectomy and identify the women most likely to benefit from CoQ10 treatment.

In conclusion, women undergoing hysterectomy with bilateral salpingectomy had a significant decline in ovarian reserve when determined by serum AMH level at 6 weeks postoperation. However, the ovarian reserve changes are not significantly different in women with CoQ10 pretreatment for 2 weeks compared with placebo. While there is a possible benefit of CoQ10 on ovarian reserve after hysterectomy, this needs to be confirmed in a more extensive population study that optimises the optimal dosage and timing of treatment.
